# Telmisartan decreases microalbuminuria in patients with type 2 diabetes mellitus following coronary artery bypass grafting

**DOI:** 10.5830/CVJA-2016-089

**Published:** 2017

**Authors:** Cevdet Furat, Riza Dogan, Gokhan Ilhan, Ekrem Bayar, İzmir Atatürk Training and Research Hospital, İzmir, Turkey, Berkan Ozpak, Hakan Kara, Şahin Bozok

**Affiliations:** Department of Cardiovascular Surgery, Faculty of Medicine, Hacettepe University, Ankara, Turkey; Department of Cardiovascular Surgery, Faculty of Medicine, Hacettepe University, Ankara, Turkey; Department of Cardiovascular Surgery, Faculty of Medicine, Recep Tayyip Erdogan University, Rize, Turkey; Department of Cardiovascular Surgery, Zonguldak Atatürk State Hospital, Zonguldak, Turkey; Department of Cardiovascular Surgery, Faculty of Medicine, Katip Çelebi University,; Department of Cardiovascular Surgery, Faculty of Medicine, Katip Çelebi University,; Department of Cardiovascular Surgery, Ada Hospital, Giresun, Turkey; Department of Cardiovascular Surgery, Faculty of Medicine, Bahcesehir University, Istanbul, Turkey

**Keywords:** telmisartan, coronary artery bypass grafting, diabetes mellitus, microalbuminuria

## Abstract

**Objective:**

This prospective study aimed to investigate the effects of the selective angiotensin receptor antagonist, telmisartan, on microalbuminuria after coronary artery bypass surgery in patients with diabetes mellitus.

**Methods:**

Patients were divided into two groups with block randomisation, using the sealed envelope technique: group T (telmisartan group) consisted of patients who received the angiotensin receptor blocking agent telmisartan 80 mg daily for at least six months in the pre-operative period; group N-T (non-telmisartan group) consisted of patients who received no telmisartan treatment. Clinical and demographic characteristics, operative and postoperative features, microalbuminuria and high-sensitivity C-reactive protein levels were compared.

**Results:**

Forty patients met the eligibility criteria for the study. The groups did not differ with regard to clinical and demographic characteristics, and operative and postoperative features. Microalbuminuria levels between the groups differed significantly in the pre-operative period, first hour postoperatively and fifth day postoperatively. C-reactive protein levels between the groups differed significantly on the fifth day postoperatively.

**Conclusion:**

Telmisartan was useful for decreasing systemic inflammation and levels of urinary albumin excretion in patients who had type 2 diabetes mellitus and had undergone coronary artery bypass surgery.

## Objective

Microalbuminuria is considered to be a marker of endothelial dysfunction and is a predictor of cardiovascular disease and mortality.^1^,^2^ Studies have implicated systemic vascular damage, extensive endothelial dysfunction, a glomerular haemodynamic state of hyperperfusion and hyperfiltration, a prothrombotic state, and a low-grade chronic inflammatory state.^3^ Microalbuminuria is also associated with several cardiovascular disease risk factors, such as hyperglycaemia, hypertension, dyslipidaemia, renal dysfunction, obesity and smoking.^4^ All of these factors contribute to the genesis of atherosclerosis.

Proteinuria is also an early marker for potentially serious renal disease in diabetics. It refers to an abnormally increased excretion rate of albumin in the urine, and is a sensitive indicator of generalised microvascular disease and a marker for vascular endothelial injury and multi-organ damage.^5^ Reduction of microalbuminuria in diabetics may retard its progression to overt diabetic nephropathy.^5^

Once microalbuminuria is present, the rate of progression to end-stage renal disease can be delayed by inhibition of the renin– angiotensin system.^6^ There is evidence that the use of agents that block the renin–angiotensin–aldosterone system, notably angiotensin receptor antagonists, may provide cardiovascular protection to diabetic patients with microalbuminuria.

Microalbuminuria increases following open-heart surgery where coronary artery bypass grafting (CABG) is utilised.^7^ CABG activates an inflammatory cascade, which may increase capillary permeability and cause microalbuminuria. The increase in capillary permeability may induce exudation of proteins from the lung capillaries into the capillary–alveolar interspace and alveoli, causing the so-called postperfusion lung, which resembles pulmonary oedema. In a recent study, Loef et al. demonstrated that CABG potentiates transient renal failure and microalbuminuria.^8^

In this study, we aimed to investigate the effects of the selective angiotensin II receptor antagonist, telmisartan, on microalbuminuria after CABG surgery in patients with diabetes mellitus.

## Methods

This observational study was approved by the local institutional review board (LUT/05/38/2006) and conducted in accordance with the amended Declaration of Helsinki and Good Clinical Practice regulations. Written informed consent was obtained from all subjects. Patients admitted to the Department ofCardiovascular Surgery of our tertiary centre between June 2006 and February 2007 who had type 2 diabetes mellitus and had undergone CABG surgery constituted the study group.

Patients were divided into two groups with block randomisation, using the sealed envelope technique: group T (telmisartan group) consisted of patients who received the angiotensin receptor blocking agent, telmisartan (Micardis®, Boehringer Ingelheim, Istanbul, Turkey) 80 mg daily for at least six months in the pre-operative period; group N-T (non-telmisartan group) consisted of patients who received neither telmisartan nor any other angiotensin receptor blockers. In both groups, no patients were using angiotensin converting enzyme inhibitors for at least six months prior to the study.

Cases with severely impaired left ventricular function, chronic pulmonary obstructive disease, severe systemic non-cardiac disease, severe renal or liver impairment, infectious diseases before surgery, malignancy, those receiving corticosteroids or other immunosuppressive treatment, and patients with stroke, inflammatory disease, and/or previous cardiac surgery, and valvular heart disease were excluded from the study.

## Surgical technique and postoperative care

Cardiac medication, including beta-adrenergic blocking agents, calcium channel blocking agents and nitrates, was continued until the morning of surgery. The same general anaesthetic drugs were used in all patients. A standard median sternotomy incision was used to expose the heart and place the internal mammary artery and saphenous vein grafts used for coronary anastomosis.

In each group, routine surgery was performed using a membrane oxygenator (Edwards Vital, Edwards Lifesciences LLC, Irvine, CA, USA), a 3-mg/kg dose of sodium heparin, 2 000 ml of Ringer’s lactate primer and a roller pump at a body temperature of 28°C. Cardiopulmonary bypass was instituted via the ascending aorta and single two-stage venous cannulation (maintained at 2.2–2.4 l/min/m^2^).

Following cross-clamping of the aorta, the heart was arrested using 10–15 cm^3^/kg cold blood cardioplegia through the aortic root and topical ice slush was continued every 20 minutes for myocardial protection. Heparin was neutralised with protamine hydrochloride (Protamin 1000; Roche, Istanbul, Turkey). The circuit was primed with 2 000 ml Ringer’s lactate.

After completion of the surgery, patients were transferred to the intensive care unit (ICU), where standard care and processes were followed until discharge. Patients were weaned from mechanical ventilation when they were haemodynamically stable, responding to verbal stimulation, and had been fully rewarmed. Patients were discharged from the ICU if they were haemodynamically stable, had normal blood gasses during spontaneous breathing, and had a satisfactory renal function.

## Outcome parameters and other variables

Smoking, obesity, hypertension, duration of diabetes, family history of coronary artery disease, pre-operative myocardial infarction, and pre-operative haemodynamic data were recorded. During the surgical procedure, haemodynamic parameters, including heart rate, mean arterial pressure, central venous pressure, arterial blood gasses and urine output were monitored. In the postoperative period in the ICU, cardiovascular and respiratory values and temperature were recorded every 15 minutes before extubation and then hourly until discharge from the ICU. The length of stay in the ICU was also recorded.

Microalbuminuria levels were studied pre-operatively, on the first hour postoperatively, and on postoperative days (POD) one and five. High-sensitivity C-reactive protein (hsCRP) levels were studied pre-operatively, and on POD 1 and 5. Patients who were considered to be in a low-cardiac output state received positive inotropic agents (dopamine or adrenaline or both). They were assessed for persistent systemic blood pressure below 90 mmHg, urinary output lower than 20 cm^3^/h, and the state of peripheral circulation was evaluated for adequate preload and optimal afterload. Urine samples were measured for microalbuminuria using Micral test sticks (Roche).

## Statistical analysis

Categorical variables were analysed with chi-squared and Fisher’s exact tests, as appropriate, in contingency tables, whereas the unpaired t-test and Mann–Whitney U-test were performed, as appropriate, for comparison of continuous variables. Comparisons for microalbuminuria and hsCRP levels in the groups were done with repeated measures of ANOVA and the Bonferroni test.

Data are expressed as means ± standard deviation. A p-value < 0.05 was considered statistically significant. All statistical analyses were performed with the Statistical Package for Social Sciences (SPSS 10.0 for Windows, SPSS, Inc., Chicago, IL). The calculation of sample size was based on a power analysis. At a power of 80% using a significance level of p < 0.05, the sample size required was 20 subjects per study group.

## Results

Forty patients met the eligibility criteria for the study. Of the 40 patients (29 males, 11 females) whose charts were reviewed, the average age was 65.0 ± 8.6 (range 40–79) years. Group T included 20 patients (15 males, 5 females) with a mean age of 65.6 ± 7.8 years, who had been using telmisartan 80 mg daily for at least six months. Group N-T included 20 patients (14 males, 6 females) with a mean age of 64.4 ± 9.5 years, who used no angiotensin receptor blocking agent prior to the operation. The groups were similar with regard to age and gender (p = 0.680 and p = 0.723, respectively).

With regard to clinical characteristics such as body mass index, smoking habit, hypertension, hyperlipidaemia, and history of myocardial infarct, the two groups did not show significant differences and were comparable (Table 1). The groups were also similar with regard to number of bypass grafts, cardiopulmonary bypass time, cross-clamp time, flow, atrial fibrillation, inotrope usage, time of endotracheal intubation and mortality rate (Table 2).

**Table 1:** Clinical and demographic characteristics of the study group

*Characteristics*	*Group T*	*Group N-T*	*p-value*
Age (years)	65.6 ± 7.8	64.4 ± 9.5	0.680
Gender (M/F)	15/5	14/6	0.723
Body mass index	28.0 ± 4.7	26.5 ± 2.8	0.234
Smoking, n (%)	11 (55)	10 (50)	0.752
Hypertension, n (%)	18 (90)	16 (80)	0.661
Hyperlipidaemia, n (%)	19 (95)	18 (90)	1.000
History of myocardial infract, n (%)	12 (60)	13 (65)	0.744

Group T = telmisartan group; group N-T = non-telmisartan group.

**Table 2:** Pre- and postoperative microalbuminuria levels

*Surgical parameters*	*Group T*	*Group N-T*	*p-value*
Number of bypasses	2.9 ± 1.0	2.9 ± 0.9	0.876
Cardiopulmonary bypass time (min)	87.4 ± 31.3	86.6 ± 20.4	0.920
Cross-clamp time (min)	52.6 ± 21.6	53.2 ± 18.5	0.925
Flow (cm^3^)	4469.0 ± 362.4	4491.0 ± 295.0	0.834
Atrial fibrillation, n (%)	4 (20)	6 (30)	0.716
Inotrope usage, n (%)	3 (15)	6 (30)	0.451
Mortality, n (%)	0	2 (10)	0.487

Group T = telmisartan group; group N-T = non-telmisartan group.

Pre-operative, first hour postoperative, POD 1 and POD 5 microalbuminuria levels were 16.5 ± 17.2, 28.5 ± 17.2, 59.0 ± 29.8 and 23.0 ± 20.0 mg/l in group T, and 30.0 ± 17.7, 51.0 ± 28.4, 75.0 ± 25.6 and 52.5 ± 27.5 mg/l in Group N-T, respectively, and there were statistically significant differences between four microalbuminiria levels in each group (p < 0.001) (Table 3). Pre-operative, first hour postoperative and POD 5 values were statistically significantly different between the groups (p = 0.018, p = 0.008 and p = 0.001, respectively) (Table 3). However, the difference in POD 1 values between the groups was at the threshold of significance (p = 0.071).

**Table 3:** Pre- and postoperative microalbuminuria levels

**	*Group T Mean ± SD*	*Group N-T Mean ± SD*	*p-value*
Pre-operative	16.5 ± 17.2	30.0 ± 17.7	0.018
Postoperative 1st hour	28.5 ± 17.2	51.0 ± 28.4	0.008
Postoperative 1st day	59.0 ± 29.8	75.0 ± 25.6	0.071
Postoperative 5th day	23.0 ± 20.0	52.5 ± 27.5	0.001

Group T: Pre-op vs 1st day: p < 0.001; pre-op vs 5th day: p = 0.036; 1st hour vs 5th day: p = 0.021; 1st day vs 5th day: p = 0.036.

Group N-T: Pre-op vs 1st day: p < 0.001; 1st hour vs 1st day: p <0.001; 1st hour vs 5th day: p < 0.001; 1st day vs 5th day: p < 0.001.

Pre-operative plasma levels of hsCRP (0.35 ± 0.17 vs 0.50 ± 0.32 mg/l) showed a trend towards significance (p = 0.069). Although POD 1 hsCRP levels (10.0 ± 2.0 vs 17.8 ± 3.9 mg/l) did not differ (p = 0.405) between the groups, a decrease in POD 5 hsCRP levels in group T (8.6 ± 2.9 vs 10.9 ± 3.2 mg/l) was statistically significant between the groups (p = 0.024) (Table 4).

**Table 4:** High-sensitivity C-reactive protein levels (mg/l).

**	*Group T Mean ± SD*	*Group N-T Mean ± SD*	*p-value*
Pre-operative	0.35 ± 0.17	0.50 ± 0.32	0.069
Postoperative 1st day	10.0 ± 2.0	17.8 ± 3.9	0.405
Postoperative 5th day	8.6 ± 2.9	10.9 ± 3.2	0.024

Group T = telmisartan group; group N-T = non-telmisartan group; SD = standard deviation.

All CABG surgeries were performed successfully. There was no repeat surgery for bleeding or peri-operative myocardial infarction in either group. The only complication was one cerebrovascular accident in the N-T group. There was no clinical or laboratory evidence of postoperative renal dysfunction in either group. Urine output during surgery and in the postoperative period did not differ between the groups. No wound infection was observed for any patient.

## Discussion

Coronary artery bypass grafting is often followed by a systemic inflammatory response. The clinical relevance of CABGrelatedsystemic inflammation varies with patients and such inflammation may be accompanied by intermittent organ dysfunction and finally, multi-organ failure, including renal and pulmonary dysfunction.^9^,^10^

In some patient groups, the effect of extracorporeal circulation is serious after open-heart surgery and it is well known that diabetic patients are frequently associated with renal and cardiovascular disease, requiring surgical and medical intensive care. Some pathophysiological mechanisms such as microalbumiuria and urinary protein over-excretion are responsible for these damaging effects in this particular group of patients.

In patients with diabetes, angiotensin II is believed to play a main role in the progression of renal damage, not only through haemodynamic effects but also non-haemodynamic effects, including stimulation of growth factors and cytokines and changes in extracellular matrix metabolism.^11^ Angiotensin II gives rise to glomerular hypertension and can alter the filtration properties of the glomerular basement membrane, leading to proteinuria.^12^-^13^ Angiotensin receptor antagonists have been shown to consistently produce favourable mortality and morbidity outcomes in endpoint trials in patients with type 2 diabetes and diabetic nephropathy.^14^-^16^

Microalbuminuria refers to the increased excretion of albumin into the urine, which is so slight that it can be detected only by sensitive immunological analysis. Microalbuminuria is measured in diabetic patients to predict incipient nephropathy. The predictive value of microalbuminuria for the expression of cardiovascular diseases has also been investigated and, in fact, is as powerful for predicting hyperlipidaemia or hypertension.^17^ Microalbuminuria also occurs in acute conditions where capillary permeability increases.

Microalbuminuria increases during major surgery such as CABG, and extracorporeal circulation activates an inflammatory cascade, which may increase capillary permeability and cause microalbuminuria. The increase in capillary permeability may induce exudation of proteins from the lung capillaries into the capillary–alveolar interspace and alveoli, causing the so-called post-perfusion lung, which resembles pulmonary oedema.

We found that telmisartan, as an angiotensin II receptor antagonist, had a significant lessening effect on microalbuminuria in type 2 diabetes patients undergoing coronary bypass surgery in our study. A significant decrease in hsCRP levels on day 5 was also noticed between the groups.

Several previous studies have shown that angiotensin receptor antagonists are effective anti-inflammatory agents, and our patients receiving telmisartan revealed decreased levels of systemic inflammation after CABG. This anti-inflammatory effect of telmisartan may help preserve postoperative renal function and also vascular endothelial function, which may also be seen after bypass surgery.

We know that renal dysfunction is a serious complication of coronary revascularisation with CABG and results in increased morbidity and mortality rates and prolonged hospital stay.^18^ The injurious action of CABG on renal function is caused by several mechanisms, including non-pulsatile perfusion and increased levels of circulating catecholamines, cytokines and free haemoglobin.^19^ These effects result in damage to the glomerular as well as tubular structures, which, in turn, may cause renal dysfunction, especially in the presence of additional risk factors.^20^-^21^

Microalbuminuria is one of the sensitive markers of increased capillary permeability and may be useful to study the systemic inflammatory response after CABG.^6^,^22^,^23^ According to previous investigations, urinary microalbuminuria increased significantly in the early postoperative period and one day after CABG.

In our study, peak increase in microalbuminuria was observed in both groups but there was no statistically significant difference (p = 0.071). These levels decreased, particularly on the fifth day in our cases, and the decrease was statistically significantly different in group T. In both groups, hsCRP increased and peaked on the first postoperative day in both groups. However, in group T, hsCRP, as one of the pro-inflammatory agents, decreased significantly on the fifth day. Therefore, the increase in acute inflammatory response was similar in both groups on the first postoperative day, and in group T, both markers had decreased by the fifth day.

Borch-Johnsen et al. showed the direct relationship between proteinuria and cardiovascular mortality rate in insulindependent diabetic patients after open-heart surgery in patients undergoing CABG.^24^ Telmisartan was also shown to reduce or normalise microalbuminuria in 34% of patients with diabetes, and in a second, smaller study including 64 hypertensive and 60 normotensive patients, to reduce the incidence of renal dysfunction. This confirmed that telmisartan reduced microalbuminuria independently of its blood pressure-lowering effects. Restoration of normal urine albumin levels has also been demonstrated by telmisartan.^25^

Our study showed that telmisartan reduced microalbuminuria, not only pre-operatively, but also after open-heart surgery. The return to baseline levels was also faster than in group N-T. Angiotensin receptor blocking agents decrease some of the postoperative acute inflammatory agents in on-pump CABG patients with diabetes mellitus by lessening the systemic consequences of renal dysfunction, and may have additional cardiovascular effects by exerting beneficial effects on endothelial tissue elsewhere in the body and within the heart in this patients group. The cardiovascular benefits of angiotensin receptor antagonists have been evaluated, not only in terms of their ability to lower blood pressure, but also on their ability to prevent strokes, cardiac events and target-organ damage.^14^,^16^

Limitations of our study are the relatively small size of our series and the lack of definite criteria for selection of patients for this study. As most coronary patients are already being treated with angiotensin receptor blocking agents, the results of our study will not have a major impact on clinical practice. Furthermore, it would have been better to test the predictivevalue of microalbuminuria on prognosis in this category of patients. However, we hope that this study will pioneer further studies on this method.

## Conclusion

Our results showed that telmisartan decreased systemic inflammation and urinary albumin excretion in diabetic patients after CABG surgery, compared to those not taking angiotensin receptor antagonists. These beneficial effects of telmisartan treatment on diabetic patients after CABG should be investigated further in prospective, randomised studies.
